# Determining the value of medical technologies to treat ultra-rare disorders: a consensus statement

**DOI:** 10.3402/jmahp.v4.33039

**Published:** 2016-10-27

**Authors:** Michael Schlander, Silvio Garattini, Peter Kolominsky-Rabas, Erik Nord, Ulf Persson, Maarten Postma, Jeff Richardson, Steven Simoens, Oriol de Solà-Morales, Keith Tolley, Mondher Toumi

**Affiliations:** 1 Institute of Public Health, Mannheim Medical Faculty, University of Heidelberg, Mannheim, Germany; 2 University of Applied Economic Sciences Ludwigshafen, Ludwigshafen, Germany; 3 Institute for Innovation and Valuation in Health Care, Wiesbaden, Germany; 4 IRCCS – Istituto di Ricerche Farmacologiche Mario Negri, Milan, Italy; 5 Interdisziplinäres Zentrum für Public Health, Universitätsklinikum Erlangen, Erlangen, Germany; 6 School of Pharmacy, University of Oslo, Oslo, Norway; 7 The Swedish Institute for Health Economics, Lund, Sweden; 8 Department of Pharmacy, University of Groningen, Groningen, Netherlands; 9 Department of Epidemiology, University Medical Center Groningen, University of Groningen, Groningen, Netherlands; 10 Centre for Health Economics, Monash University, Clayton, Victoria, Australia; 11 Department of Pharmaceutical and Pharmacological Sciences, Katholieke Universiteit (KU) Leuven, Leuven, Belgium; 12 Health Innovation Technology Transfer (HITT), Barcelona, Spain; 13 Tolley Health Economics Ltd, Buxton, Derbyshire, Great Britain; 14 Public Health Department – Research Unit EA 3279, Faculty of Medicine, Aix Marseille University, Marseille, France

**Keywords:** orphan drugs, health technology assessment, economic evaluation, cost-effectiveness, social cost value analysis, multicriteria decision making

## Abstract

**Background:**

In most jurisdictions, policies have been adopted to encourage the development of treatments for rare or orphan diseases. While successful as assessed against their primary objective, these policies have prompted concerns among payers about the economic burden that might be caused by an annual cost per patient in some cases exceeding 100,000 Euro. At the same time, many drugs for rare disorders do not meet conventional standards for cost-effectiveness or ‘value for money’. Owing to the fixed (volume-independent) cost of research and development, this issue is becoming increasingly serious with decreasing prevalence of a given disorder.

**Methods:**

In order to critically appraise the problems posed by the systematic valuation of interventions for ultra-rare disorders (URDs), an international group of clinical and health economic experts was convened in conjunction with the Annual European ISPOR Congress in Berlin, Germany, in November 2012. Following this meeting and during subsequent deliberations, the group achieved a consensus on the specific challenges and potential ways forward.

**Results:**

The group concluded that the complexities of research and development for new treatments for URDs may require conditional approval and reimbursement policies, such as managed entry schemes and coverage with evidence development agreements, but should not use as justification surrogate end point improvement only. As a prerequisite for value assessment, the demonstration of a minimum significant clinical benefit should be expected within a reasonable time frame. As to the health economic evaluation of interventions for URDs, the currently prevailing logic of cost-effectiveness (using benchmarks for the maximum allowable incremental cost per quality-adjusted life year gained) was considered deficient as it does not capture well-established social preferences regarding health care resource allocation.

**Conclusion:**

Modified approaches or alternative paradigms to establish the ‘value for money’ conferred by interventions for URDs should be developed with high priority.

This paper documents an international expert consensus that emerged from debate during a face-to-face meeting in Berlin, Germany, on 8 November 2012, followed by an exchange of thoughts by phone and mail on two draft versions, describing the results of the workshop. A prefinal version of the document was completed by 19 July 2013, and the consensus was confirmed and refined at subsequent workshops of the expert group in Dublin, Ireland, on 7 November 2013 and in Amsterdam, Netherlands, 13 November 2014.

The reasoning underlying the consensus statement has been published separately in a peer-reviewed paper, which provides an extensive overview and discussion of important references on the subject (1). In the following sections, we present the original consensus statement with very minor edits only. The edits do not change the material content but were introduced with the objective to make the paper more intelligible, incorporating helpful advice from two anonymous reviewers.

## Background and problem statement

In the United States and the European Union, as well as in Japan, Australia, and some other jurisdictions, legislation has been adopted to encourage the development of treatments for rare or orphan diseases. Under this legislation, developers and manufacturers of so-called orphan drugs used to treat orphan diseases benefit from a range of incentives, including reduced or waived licensing fees, extended market exclusivity periods, and, in the United States and Japan, tax relief on development costs.

In theory, there are no distinct (sub-) categories of rare and ultra-rare disorders (URDs) and treatments. Increasing rarity of a condition merely represents the end of a continuum, just like increasing severity and increasing comorbidities represent continuous, not discrete phenomena. For policy makers, it may nevertheless be pragmatic to define different categories of disorders and interventions, irrespective of the (absence of) theoretical merits of such an approach.

The term ‘orphan disorders’ has been defined by US and EU legislation. In the United States, these are disorders with a prevalence of fewer than 200,000 affected persons; in the European Union, prevalence must be less than 5 per 10,000 (or less than 0.05%) of the population. Currently, no official definition of ‘ultra-orphan disorders’ has been adopted globally. Rather, this informal subcategory was introduced by the National Institute for Health and Care Excellence (NICE) (formerly the Institute for Health and Clinical Excellence and the National Institute for Clinical Excellence), who applied it to drugs with indications for conditions with a prevalence of less than 1 per 50,000 persons (2). The definition, albeit no less arbitrary than the definitions used for ‘orphan disorders’, corresponds to the even more restricted prevalence criteria adopted by England's Advisory Group for National Specialist Services, assigned to review technologies for URDs that treat fewer than 500 persons in England (i.e., approximately 1 in 100,000 of the English population).

**Fig 1:**
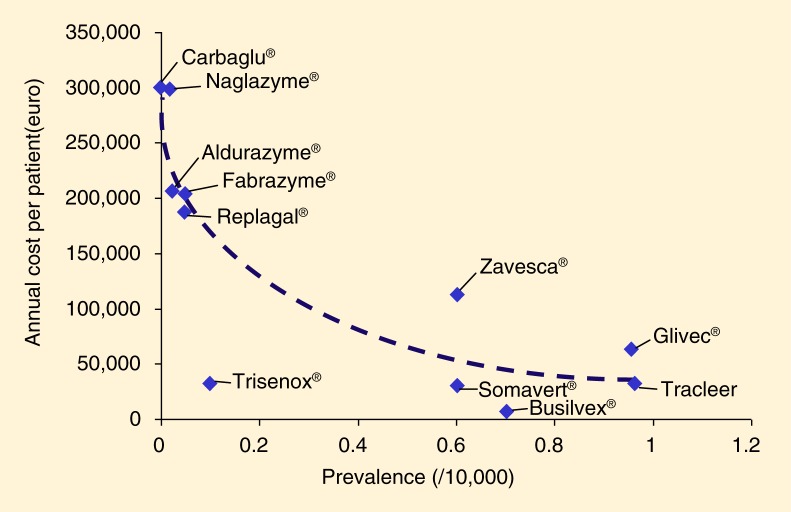
Increasing acquisition cost per patient with decreasing prevalence, as a result of fixed (i.e., largely volume-independent) research and development expenditures. [Adapted from Schlander and Beck (3, p. 1290); based on original data from Alcimed (4)].

It is easy to see that many drugs developed to treat URDs will not meet the cost-effectiveness thresholds stipulated by some official regulatory bodies such as NICE, that is, not to exceed a cost of £20,000–£30,000 per quality-adjusted life year (QALY) gained (Table 1). Given the largely fixed (i.e., independent from sales volume) costs of research and development, it seems plausible that this challenge will increase in relevance with decreasing prevalence rates, especially with drugs developed to treat very small patient populations (cf. Fig. 1, below).

**Table 1:** Preliminary cost per QALY ICER estimates by NICE (2005) (2), illustrating the mismatch between utra-orphan drug cost and conventional cost-effectiveness benchmarks (as, e.g., adopted by NICE, from £20,000 to £30,000 per QALY gained)

Condition	Prevalence (England)	Product	ICER (preliminary estimated cost in GBP per QALY)
M. Gaucher Type I and III	270	Imiglucerase (Ceredase^R^)	391,200
MPS Type 1	130	Laronidase (Aldurazyme^R^)	334,900
M. Fabry	200	Agalsidase beta (Fabrazyme^R^)	203,000
Hemophilia B	350	Nonacog alpha (BeneFIX^R^)	172,500
M. Gaucher Type I	270	Miglustat (Zavesca^R^)	116,800

ICER, incremental cost-effectiveness ratios; QALY, quality-adjusted life years; NICE, National Institute for Health and Care Excellence.

The introduction of an ultra-orphan category by NICE can thus be interpreted as a defensive move, responding to political and public pressures that NICE experienced as a reaction to negative appraisals. It also can be seen as an attempt to protect NICE's evaluation framework, while at the same time recognizing that this framework (in an unspecified way) ‘does not work’ for ultra-orphan drugs.

A similar move by NICE was the introduction of a second special category, so-called end-of-life treatments. The need to create exceptions may point to deeper issues affecting the generalizability of the ‘logic of cost-effectiveness’ as adopted by NICE. It has been argued that at least some of these issues may indeed relate to well-understood deficiencies of the logic of cost-effectiveness (or the ‘extra-welfarist proposition’, the foundations of which will be discussed later).

Apparently, there is a serious mismatch between reimbursement policies based on the logic of cost-effectiveness with cost-per-QALY benchmarks, on the one hand, and international policies designed to encourage research and development into rare and URDs and their effective treatment, on the other hand. As such, there appears to be an unmet need for a coherent value framework reflecting all attributes of health technologies deemed relevant by the public (‘social preferences’), while at the same time remaining consistent with prior normative commitments as entailed by institutional and legal traditions. Such a framework should also enable decision makers to effectively address the specific challenges that are posed by health technology assessments (HTAs) of interventions for diagnosis and treatment of rare and URDs, combining fair access to effective interventions (for patients) with incentives for research, development, and ‘innovation’ (for manufacturers) and a set of clear principles for setting limits (for policy makers and payers).

## Objectives and methods

In order to address this situation, the not-for-profit Institute for Innovation and Valuation in Health Care (Wiesbaden, Germany) convened an international expert workshop in Berlin, Germany, on 8 November 2012.^1^ Organization of the 1-day workshop was supported by two biopharmaceutical firms, Alexion (Cheshire, CT, USA) and BioMarin (San Rafael, CA, USA), under an unrestricted educational grant policy.

The objectives of the workshop were as follows:

1. To review the challenges that arise when applying conventional HTA methodologies to medical technologies for ultra-rare diseases
2. Given these challenges, to seek expert agreement on the need for (improved or) alternative evaluation methods, ideally in the form of a consensus statement
3. In light of this analysis, to initiate discussion of improved or alternative evaluation methods, including the advantages and disadvantages of different options and possible ways forward

The agreed workshop agenda (Appendix II) adhered closely to the objectives set out above.

In order to facilitate an open exchange of ideas and views in the process, the workshop participants agreed to comply with the Chatham House Rule: ‘When a meeting, or part thereof, is held under the Chatham House Rule, participants are free to use the information received, but neither the identity nor the affiliation of the speaker(s), nor that of any other participant, may be revealed’.

After the workshop, two consecutive draft summary documents were distributed to the participating experts, whose comments were integrated in an iterative process, leading to the final consensus document presented here. The consensus was formally adopted at subsequent meetings of the expert group in Dublin, Ireland, on 7 November 2013, and in Amsterdam, Netherlands, on 13 November 2014.

Workshop participants agreed that the project should begin with a situation analysis in order to establish common ground for future deliberation by the expert panel. To this end, various levels of analysis were distinguished, namely a focus on the following:

1. The *principles* underlying the current evaluation framework.
2. The actual evaluation *policies* implemented by HTA agencies and regulatory bodies (primarily those concerned with pricing and reimbursement decisions).
3. Evaluation *practice* when principles and policies are applied to real-world problems. In particular, the third level would have to include case studies, including cases where existing regulation has been potentially misused.

The group agreed that discussion should initially focus on fundamental principles, because policy implementation and evaluation practice (although clearly relevant dimensions) represent hierarchically lower levels of analysis. Review of the latter should be done with reference to a set of high-level guiding principles agreed on prior to moving to application.

## Definitions

While recognizing the somewhat arbitrary nature of this cut-off criterion, the expert group agreed to focus on medical technologies targeting URDs (with a prevalence of less than 1 per 50,000), that is, to exclude from further analysis the following related but different subject areas:

1. Orphan disorders with a prevalence of less than 5/10,000 (or less than 1/2,000) but more than 1/50,000
2. Cancer medicine (given its distinct characteristics, including the frequently observed gradual expansion of indications, for example by moving treatments from third or fourth line to second line, combined, or adjuvant use in early stage disease)
3. The specific challenges posed by emerging concepts of ‘personalized medicine’
4. Also, for the time being (cf. above), abusive commercial ploys such as ‘indication slicing’ and other strategic games played by some manufacturers^2^

Further characteristics of URDs under consideration should include conditions that

- Are *severe*.
- Are *chronic*.
- Represent *clearly defined biological entities* (i.e., are *not* ‘created’ by artificial ‘slicing’ of a biologically much broader and more prevalent indication).
- Hence, are associated with a broadly accepted *high unmet medical need*. However, the absence of alternative treatment options was not considered a necessary defining condition of an URD, as the broader criterion of ‘high unmet medical need’ was believed to better capture the underlying rationale.

The subjects of analysis were specific (unique) condition/treatment pairs fulfilling the criteria listed above, combined with a clear biological rationale. The typical case the workshop participants had in mind were treatments that are effective for one URD only (such as enzyme replacement therapies for hereditary lysosomal storage disorders). The panel shared the view that certain adjustments would probably be necessary when one drug works in more than one URD indication. However, these adjustments were considered likely to be of a rather technical nature and hence were not explored in detail at the workshop, as its primary focus was on discussion of underlying fundamental evaluation principles.

## Results: specific challenges

While recognizing the continuum (instead of an arbitrary prevalence threshold) related to increasing ‘rarity’, the group of experts agreed that, in principle, a number of typical challenges must be expected when dealing with interventions for URDs. The most serious ones fall into one or both of the following categories: 1) the need to establish evidence of clinical effectiveness and 2) the need to demonstrate ‘value for money’.

### Establishing evidence of clinical effectiveness

Developing treatments for URDs is a more challenging, complex, and sometimes risky endeavor than developing treatments for more common diseases, as

- Less clinical/medical research is often available for URDs, resulting in a limited clinical understanding.
- There is usually a very small number of physicians with specialized expertise, who are based in a few specialized centers.
- Unusual difficulties exist to produce robust clinical evidence, for example, because of limited understanding of the natural history of URDs and because of the often-limited availability of validated instruments to measure disease severity and progression.
- This, combined with difficulties to generate a large volume of evidence for URDs based on randomized clinical trials, may lead to higher levels of uncertainty surrounding effect size estimators.
- Significant hurdles exist when trying to identify and accurately diagnose patients with URDs.
- Because the small number of patients are often geographically dispersed, multiple clinical trial sites must be established for only a few patients.
- Ongoing postmarketing requirements, including registries and risk management plans, must be created and maintained globally for only a small number of patients.
- As a consequence, in a significant number of cases, the safety and efficacy profiles of orphan drugs have been incomplete, and often marketing authorizations were based on small-scale studies addressing surrogate end points only (6).

The expert group recognized the need for ongoing research and development (R&D) for highly innovative and lifesaving products for URDs, in order to increase clinical disease understanding and produce robust evidence on the clinical effectiveness of interventions (‘technologies’ in the broadest sense).

### Establishing ‘value for money’ (efficiency)

Further challenges are related to but extend beyond the sphere of evidence generation to demonstrate clinical effectiveness of technologies. These challenges are economic in nature; they concern the efficiency or ‘value for money’ offered by URD treatments:

- Across health care systems, there is a marked heterogeneity regarding institutional arrangements. This is mirrored by the situation that currently established methodologies to determine ‘value for money’ vary internationally, with a stronger utilitarian tradition (as, e.g., in England) generally leading to a higher acceptance of ‘efficiency-first’ evaluation principles, whereas stronger emphasis on a rights-based approach (and a corresponding legal tradition, as e.g., in some continental European countries such as France and Germany) has led to a stronger reliance on approaches based on unmet medical need and on evidence of comparative clinical effectiveness for the allocation of health care resources.
- In applied health economics – in contrast to neoclassical welfare economics – health outcomes (rather than ‘utility’) are usually considered to be the appropriate benefit for evaluation. This ‘extra-welfarist’ view has been gaining popularity because of the widespread belief that basic necessities ‘such as life, health, and citizenship […] should be distributed less unequally than the ability to pay for them’ (J. Tobin (1970), p. 263 (7)). Usually this currently prevailing health economic evaluation paradigm is accompanied by the assumption that the objective of collectively financed health schemes ought to be simple maximization of the aggregate health gain produced for the population covered by the scheme. If and when health gains are measured in terms of QALYs, extra-welfarism then translates into QALY maximization, a normative hypothesis that has been endorsed by extra-welfarists on grounds of an alleged ‘consensus in the literature’ (G.W. Torrance (2006), p. 1071 (8)).
- From there it is possible and straightforward to establish a ranking of medical interventions based on their efficiency as defined by their incremental cost per QALY gained (sometimes called *QALY league tables*, based on incremental cost-effectiveness ratios, ICERs), implying a presumably increasing social desirability of services associated with decreasing ICERs. In practice, this approach translates into the adoption of some sort of a benchmark for the maximum allowable cost per QALY, which may be interpreted as the social willingness to pay for, or the shadow price of, a QALY. Interventions meeting this benchmark criterion will then be deemed ‘efficient’ given a resource constraint.
- Notwithstanding claims of distributive neutrality (‘a QALY is a QALY is a QALY, regardless of who gains or loses it’), however, this approach implies considerable constraints on the preferences to be taken into account. Any contextual variable(s) – apart from individual health gain – potentially influencing the social desirability of (and hence the social willingness to pay for) health services would necessarily violate the basic assumption that all QALYs are created equally.
- If there were other objectives beyond the maximization of population health (which represents the goal of allocative efficiency), such as the wish to be treated with dignity and respect or concerns about equity and fairness (e.g., with regard to equality of access to care, equal access for equal need, etc.), these quite obviously would either result in differential cost-per-QALY benchmarks as a function of these concerns. Alternatively, they might even require an entirely new evaluation paradigm. This issue has been described using the notion of *horizontal equity* (i.e., the equal treatment of equals) versus *vertical equity* (i.e., the unequal but equitable treatment of unequals, which would imply differentiation based on characteristics of the respective diseases and the patient groups afflicted with them).
- As noted in the introduction, many interventions for rare diseases and URDs are unlikely or altogether unable to meet standard cost-per-QALY benchmarks. Hence, there is a need to examine the range of normative and empirical issues surrounding the application of the extra-welfarist logic of cost-effectiveness (as a criterion for *allocative efficiency*) for the prioritization of health care programs. It is noteworthy that, in an attempt to escape from contentious interpersonal comparisons, politicians and health care policy makers in some jurisdictions, such as the United States and Germany, have deliberately decided to refrain from the computation of cost per QALY gained, in essence restricting themselves to the evaluation of comparative effectiveness PCORI in the United States and GBA in Germany as a result of the most recent health care reform acts) or, at best, of *technical efficiency* (e.g., methods guidance by IQWiG in Germany, designed to avoid using the same benchmarks for all interventions, across different disorders).
- With either approach, there remains the need to establish fair boundaries with regard to coverage (reimbursement) and pricing and, as an immediate consequence, with regard to access to medical technologies, given the limited willingness of the public to be taxed (or the limited social willingness to pay for health insurance).

### Social preferences and valuation

Specific normative as well as technical problems arise when traditional HTAs include cost–utility analyses, with QALYs as a measure of health-related outcomes (and their individual valuation) for URDs:

- *Social value*, as indicated by the social preferences of the population covered by a national health scheme (NHS) or an insurance plan, is not identical to some kind of an aggregate of individual utility (which is usually assumed to be approximated sufficiently well by the strength of individual preferences, usually derived either from patients or, more often, from a representative sample of the general population).
- Rather, *social preferences* notably include equity concerns and a ‘sharing’ perspective:
- In light of the observations above, QALYs, conceptualized as a preference-based measure of individual health-related outcomes combining quality and length of life, seemingly fail to capture the full value of URD technologies. Hence they need to be complemented by or replaced with alternatives that include societal preferences, such as concerns for equity in access to treatment.
- Current (cost-per-QALY) ICER thresholds used for cost-effectiveness (or more precisely, cost–utility) analysis are largely arbitrary and inappropriate when used to evaluate URD technologies; their application may lead to positively unethical conclusions that might deprive patients with URDs any chance of access to effective care, thus conflicting with fairness- and rights-based considerations.
- The very existence of such thresholds (outside the confines of the narrow extra-welfarist framework) depends on the validity of the QALY maximization hypothesis, whereas systematic reviews of the literature have convincingly shown that this assumption is ‘descriptively flawed’, that is, these thresholds do not capture well-established social preferences beyond the quasi-utilitarian (health outcomes) maximization principle (which, by design, is ‘distribution-blind’).
- Attempts to apply modifiers to account for severity of disease (so-called ‘*equity*’ or ‘*severity weights*’) in economic assessments of technologies for URDs have not fully reflected the large number of contextual variables and cannot solve the underlying issues with regard to fair chances to have access to effective treatment.

### Social preferences and costs

1. Importantly, studies further suggest that the *importance of costs* may be overstated by conventional health economic evaluations, since cost-minimization, cost-effectiveness, cost–utility, and cost–benefit analyses, by definition, focus significantly on cost. In contrast to this, the public does not appear to be well prepared to deny patient treatment merely on the basis of cost – which apparently constitutes a social preference related to some kind of fairness or rights-based reasoning similar to the dislike of ‘all-or-nothing’ decisions but does not necessarily imply valuing ‘rarity’ *per se*.
2. *Costs per patient* for URD treatment will necessarily tend to be (much) higher than cost per patient for more common disorders, given the R&D issues delineated above, in combination with the fixed-cost nature of R&D expenses, logistical challenges, and (sometimes) manufacturing complexities. As to cost, most technologies for URDs have a limited overall budget impact, particularly when weighed against the clinical and societal benefits of such treatments:
3. Although this observation is usually true for individual treatments, the combined budgetary impact of the health service costs for many URDs may be more profound.^3^
4. However, URD treatments represent only a presumably small part of the entire group of orphan drugs.

## Discussion: potential ways forward

Collectively, the findings and observations summarized above underscore the need for an evaluation paradigm capturing and reflecting social preferences in a better way than the conventional logic of cost-effectiveness, with potentially far-reaching implications for the evaluation of URDs.

### Evidence of clinical effectiveness

The starting point of any value analysis can only be clinical benefit. In their comprehensive review of the first decade of orphan drug legislation in the European Union, Roberta Joppi and colleagues (2013) found that many orphan drugs were approved with evidence of surrogate end point effects only (6). In the absence of sufficiently strong evidence for some minimum significant benefit, however, the basis is lacking for any robust value determination.

While recognizing the challenges associated with developing clinical interventions for URDs, the panel agreed that evidence for improvement of surrogate end points only should be no more than an interim attitude, providing a basis for provisional approval and reimbursement, in order to ensure patients fast access to new technologies. It could be linked to managed entry schemes such as ‘coverage with evidence development’ agreements in order to incentivize further research. Even at a prevalence rate of a given condition as low as 1/50,000 (the URD qualifier), there will be about 10,000 patients in Europe. Thus it should be possible to set up multinational randomized controlled trials, including between 500 and 1,000 patients, designed to show relevant clinical end point benefit. If necessary, such trials might be supported by the not-for-profit European Clinical Research Infrastructures Network initiative devoted to promote multinational studies.

### Perspectives on cost

As stated earlier, the cost per patient will tend to be higher with decreasing prevalence. Budget impact, however, can be looked upon in various different ways.

1. One prevalent view (consistent with the *efficiency-first approach* advocated by conventional health economics) is that budget impact should not be relevant to coverage decisions, which ought to be based on incremental cost-effectiveness. For example, NICE has taken the position that budget impact analyses should not form part of the decision-making process; rather, they should be used as a tool aiding UK Regional Health Authorities in implementing NICE guidance locally.
2. Given the ‘silence of the lambda’ (i.e., ICERs by design providing no information on the dimension of a program, as the sizes of the numerator and the denominator cancel out (10)), health care policy makers are concerned with the budget impact of adopting a technology (consistent with the notion of affordability), and methods have been proposed by health economists for how it might be possible to combine incremental cost-effectiveness and budget impact into one metric.
3. If a *social value perspective* (instead of a focus on individual utility) were to be adopted in a consistent manner, then there could be simultaneous implications for the definition of social opportunity cost (or value foregone), with the social value being driven by the existence of a program (i.e., for example the value people might attach to living in a society that does not simply abandon certain groups of patients who are unfortunate enough to suffer from a high-cost illness) and opportunity cost by its budgetary impact. This would obviously shift the focus from cost per patient to cost on the program level, which indeed reflects the perspective of a real-world decision maker (11).
4. Finally, a more pragmatic approach might combine rights-based thinking in terms of a desire to offer fair chances to receive effective treatment also to patients with URDs with the realities of pharmaceutical R&D and its fixed-cost structure, resulting in the implementation of price/volume trade-offs as realized, for example, in France.

### Valuation principles

Potential evaluation principles better (compared to the logic of cost-effectiveness using cost-per-QALY benchmarks) reflecting the public's social preferences may include, at different levels of analysis:

- A method combining traditional cost-effectiveness with budget impact analysis
- Cost–value analysis by means of adjusting cost-per-QALY benchmarks according to multiple contextual variables
- Cost–value analysis using the person trade-off method (12)
- Cost–value (or social utility) analysis using the relative social willingness to pay instrument
- A multicriteria decision analysis framework
- Using ‘capability-adjusted life years’ instead of QALYs as a measure of benefit
- Using healthy-year equivalents as a measure of benefit
- Applying different perspectives on the measurement of costs
- On the methodological level, discrete choice experiments, conjoint analysis and/or analytical hierarchy process techniques measuring and integrating benefits from a patient's perspective

All of these should be rigorously assessed for their potential to improve on the currently predominant standard, which is still represented by cost–utility analysis (1). Given the limitations of the conventional approach, the strengths and weaknesses of each of the alternatives should be explored with high priority.

## Footnotes

1 For a complete list of workshop participants, see Appendix I.

2 For a discussion of some of the most prevalent commercial strategies, see for example W. Hughes-Wilson et al. (2012) (5).

3 This will be especially relevant if and when orphan drugs, cancer treatments, and recent developments described as ‘personalized medicine’ are taken into account, too; however, the focus of the present discussion is specifically on the extreme case of URDs – for orphan drugs, recent estimates of budget impact seem to converge at 3–3.5% of the drug budget in many European countries (9).

## Appendix I: Meeting attendees

1. Experts (Panelists)
  - Silvio Garattini
    Professor of Pharmacology and Foundation Director, Istituto di Ricerche Farmacologiche Mario Negri, IRCCS (Milan, Italy)
  - Peter Kolominsky-Rabas
    Professor, University of Erlangen (Erlangen, Germany)
  - Erik Nord
    Professor, University of Oslo and Norwegian Institute of Public Health (Oslo, Norway)
  - Ulf Persson
    CEO, Swedish Institute for Health Economics (Lund, Sweden)
    Professor, Lund University (Lund, Sweden)
  - Maarten Postma
    Professor, University of Groningen (Groningen, The Netherlands)
  - Jeff Richardson
    Professor and Foundation Director, Centre for Health Economics, Monash University (Melbourne, Victoria, Australia)
  - Michael Schlander
    Founder and Chairman, Institute of Innovation and Valuation in Health Care (Wiesbaden, Germany)
    Professor, University of Applied Economic Sciences Ludwigshafen (Ludwigshafen, Germany)
    Professor, Mannheim Institute of Public Health, Heidelberg University (Mannheim, Germany)
  - Steven Simoens
    Professor, Leuven University (Leuven, Belgium)
  - Oriol de Solà-Morales
    Director, Pere Virgili Institute for Health Research (Tarragona, Spain)
  - Keith Tolley
    CEO, Tolley Health Economics (Derbyshire, UK)
  - Mondher Toumi
    Professor, University of Lyon (Lyon, France)
2. Guests
  - Mohit Jain
    Director, Market Access and Public Policy EUMEA, BioMarin (London, England)
  - Sarah Pitluck
    Senior Director, Global Pricing and Reimbursement, Alexion Pharmaceuticals (Washington, DC, USA)
  - Urbano Sbarigia
    Associate Director, EMEA Pricing and Reimbursement, Alexion Pharmaceuticals (Brussels, Belgium)
  - Ruth Suter
    Senior Director, Market Access North America, BioMarin (San Rafael, CA, USA)

## Appendix II: Workshop agenda

*Berlin, Germany, 8 November 2012*

**Table:**

9:00 a.m.	Welcome and introductions
9:30 a.m.	Overview and discussion: background on development of technologies for ultra-rare diseases
10:00 a.m.	Overview and discussion: technical problems with use of conventional health technology assessments for technologies for ultra-rare diseases
11:30 a.m.	Identify areas of agreement on potentially inappropriate use of conventional health technology assessments for technologies for ultra-rare diseases
12:15 p.m.	Discussion: potential alternatives to evaluate technologies for ultra-rare diseases
2:00 p.m.	Prioritize potential alternative evaluation approaches for further discussion and next steps
3:00 p.m.	Workshop concludes

## Appendix III: Abbreviations used

GBA: Gemeinsamer Bundesausschuss (Joint Federal Committee, the official HTA agency in Germany, in charge of appraisal determinations)

HTA: health technology assessment

ICER: incremental cost effectiveness ratio

InnoVal-HC: Institute for Innovation & Valuation in Health Care, a not-for-profit organization in Wiesbaden, Germany

IQWiG: Institut für Qualität und Wirtschaftlichkeit im Gesundheitswesen (Institute for Quality and Efficiency in Health Care, an official HTA agency in Germany, tasked with technology assessments)

ISPOR: International Society for Pharmacoeconomics and Outcomes Research

NHS: National Health Service (UK), or national health scheme (generic)

NICE: National Institute for Health and Care Excellence in London, England, established in 1999 as official body in charge of HTAs for the UK NHS

PCORI: Patient-Centered Outcomes Research Institute, an independent nonprofit, nongovernmental organization located in Washington, DC, authorized by the US Congress in 2010.

QALY: quality-adjusted life year

R&D: research and development

URD: ultra-rare disorder
